# Neuronal SNARE complex assembly guided by Munc18‐1 and Munc13‐1

**DOI:** 10.1002/2211-5463.13394

**Published:** 2022-03-22

**Authors:** Shen Wang, Cong Ma

**Affiliations:** ^1^ 12443 Key Laboratory of Molecular Biophysics of the Ministry of Education College of Life Science and Technology Huazhong University of Science and Technology Wuhan China

**Keywords:** Munc13, Munc18, SNARE complex assembly, SNAREs, synaptic exocytosis, synaptic vesicle fusion

## Abstract

Neurotransmitter release by Ca^2+^‐triggered synaptic vesicle exocytosis is essential for information transmission in the nervous system. The soluble N‐ethylmaleimide sensitive factor attachment protein receptors (SNAREs) syntaxin‐1, SNAP‐25, and synaptobrevin‐2 form the SNARE complex to bring synaptic vesicles and the plasma membranes together and to catalyze membrane fusion. Munc18‐1 and Munc13‐1 regulate synaptic vesicle priming via orchestrating neuronal SNARE complex assembly. In this review, we summarize recent advances toward the functions and molecular mechanisms of Munc18‐1 and Munc13‐1 in guiding neuronal SNARE complex assembly, and discuss the functional similarities and differences between Munc18‐1 and Munc13‐1 in neurons and their homologs in other intracellular membrane trafficking systems.

## Graphical abstract

Munc18‐1 and Munc13‐1 regulate synaptic vesicle exocytosis via orchestrating neuronal SNARE complex (syntaxin‐1, SNAP‐25, and synaptobrevin‐2) assembly. In this review, we summarized recent advances towards the molecular mechanisms of Munc18‐1 and Munc13‐1 in guiding neuronal SNARE complex assembly and discussed the similarities and differences between Munc18‐1 and Munc13‐1 in neurons and their homologs in other intracellular membrane trafficking systems.

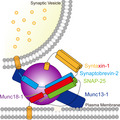

**Abbreviations**
CAPS: Ca^2+^‐dependent activator protein for secretion
CATCHR: complex associated with tethering containing helical rods
COG: conserved oligomeric Golgi
CORVET: class C core vacuolar/endosomal tethering
Dsl1: dependent on Sly1‐20
GARP: golgi associated retrograde protein
HOPS: Homotypic fusion and vacuolar protein sorting
NSF: N‐ethylmaleimide sensitive factor
SM: Sec1/Munc18‐1
SNARE: soluble N‐ethylmaleimide sensitive factor attachment protein receptor
TRAPP: transport protein particle

Neurotransmitter release by Ca^2+^‐triggered synaptic vesicle exocytosis is an exquisitely regulated process essential for information transmission in the nervous system [1, 2, 3]. Under resting conditions, most newly formed and recycled synaptic vesicles are stored in the cytoplasm of the nerve terminal. A subset of synaptic vesicles can be attached to specialized sites at the presynaptic active zones, where a number of multidomain proteins constitute a scaffold platform to mediate vesicle tethering and docking [4, 5]. To achieve fast exocytosis, docked vesicles require maturation into a ‘priming’ state that involves a population of ‘ready‐for‐fusion’ vesicles corresponding to the readily releasable pool (RRP) [6, 7, 8]. When an action potential arrives at the axon terminal, Ca^2+^ influx triggers primed vesicles to fuse with the presynaptic membrane in the millisecond timescale [9, 10].

The core release machinery governing synaptic vesicle exocytosis consists of components that belong to protein families involved in most types of intracellular membrane trafficking systems and with conserved roles in membrane fusion, including the AAA+ ATPase N‐ethylmaleimide sensitive factor (NSF), soluble NSF adaptor proteins (SNAPs), the SNAP receptors (SNAREs), Sec1/Munc18‐like (SM) protein Munc18‐1, and complex associated with tethering containing helical rods (CATCHR) family protein Munc13s [11, 12, 13]. In addition, the release machinery contains specialized components such as synaptotagmins and complexins, whose functions and mechanisms in regulating Ca^2+^‐triggered synaptic vesicle fusion have been reviewed previously [14, 15, 16]. The neuronal SNAREs serve as the engine of the release machinery, as their assembly into the SNARE complex provides energy for membrane bridging and fusion [17, 18]. Tight control and regulation of SNARE complex assembly by regulatory components are the prerequisites for synaptic vesicle fusion occurring at the right place, at the right time, and with the right probability.

Priming of synaptic vesicles is believed to be a hallmark of synaptic vesicle exocytosis, which involves activation and regulation of SNARE complex assembly. Recent advances toward the structural insights, intermolecular interactions, and functional properties of the priming components shed new light on understanding the molecular mechanism of synaptic vesicle exocytosis. In this review, we summarize recent progress on crucial priming apparatus that includes the SNAREs, Munc18‐1, and Munc13s—focusing on the functional properties and molecular mechanisms of Munc18‐1 and Munc13‐1 in organizing neuronal SNARE complex assembly—and discuss the functional similarities and differences between Munc18‐1 and Munc13s in neurons and their homologs in other intracellular membrane trafficking systems.

## The neuronal SNAREs

**Fig. 1:**
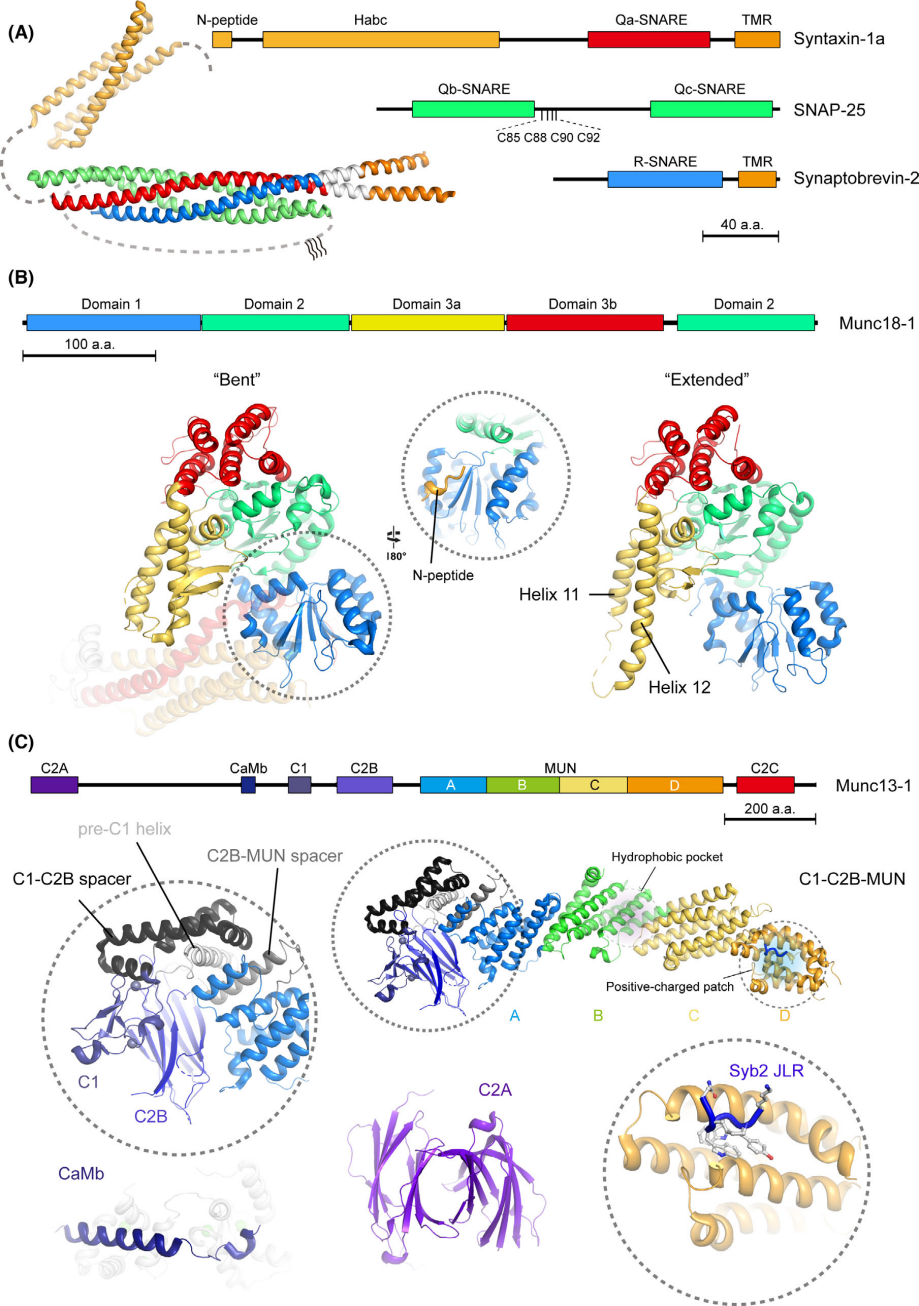
Structure illustration of the neuronal SNAREs, Munc18‐1, and Munc13‐1. (A) Crystal structure and domain illustration of the neuronal SNARE complex. The structure models are fetched from the protein data bank (PDB) by entries of 3HD7 (helical extended neuronal SNARE complex) and 1EZ3 (H_abc_ domain of syntaxin‐1). The SNARE motif of syntaxin‐1 (Q_a_‐SNARE, residues 191−253), SNAP‐25 (Q_b_‐ and Q_c_‐SNARE, residues 19−81 and 140−202), and synaptobrevin‐2 (R‐SNARE, residues 29−87) are colored in red, green, and blue, respectively. The N‐peptide (residues 1−10) and H_abc_ (residues 26−146) of syntaxin‐1 are colored in bright orange. Transmembrane domains (TMRs) of syntaxin‐1 (residues 266−288) and synaptobrevin‐2 (residues 95−114) are colored in orange. The juxtamembrane linker regions (JLRs) of syntaxin‐1 and synaptobrevin‐2 are colored in light gray. The S‐palmitoyl cysteines of SNAP‐25 are indicated as C85, C88, C90, and C92, respectively. Missing densities in the structural model are supplied by dashed lines. (B) Structural illustration of *Rattus norvegicus* Munc18‐1 with two conformations. The subdomains of Munc18‐1 are colored in blue (domain 1, residues 4−134), green (domain 2, residues 135−245 and 490−592), yellow (domain 3a, residues 246−360), and red (domain 3b, residues 361−479), respectively. Left panel displays the ‘bent’ conformation of Munc18‐1 that binds to syntaxin‐1 (PDB entry: 3C98), where the helix 11 and 12 of domain 3a are folded back. Inset shows the binding between N‐peptide (bright orange) of syntaxin‐1 and domain 1 of Munc18‐1. Right panel displays the ‘extended’ conformation of Munc18‐1 (PDB entry: 3PUJ), in which helix 11 and 12 are outstretched. (C) Structural illustration of *Rattus norvegicus* Munc13‐1. The domains and subdomains of Munc13‐1 are colored in purple (C_2_A, residues 1−97), dark blue (calmodulin‐binding domain, CaMb, residues 459−492), navy blue (C_1_, residues 566−616), purple blue (C_2_B, residues 683−820), blue (MUN‐A, residues 859−1005), green (MUN‐B, residues 1006−1167), yellow (MUN‐C, residues 1168−1318), bright orange (MUN‐D, residues 1319−1531), and red (C_2_C, residues 1558−1685), respectively. The structural models of C_1_‐C_2_B‐MUN fragment (PDB entry: 5UE8 and 6A30), C_2_A (dimer form, PDB entry: 2CJT), and CaMb (binding with calmodulin, PDB entry: 2KDU) are fetched from PDB. Two functional regions of the MUN domain, that is, the hydrophobic pocket (binds to syntaxin‐1) and the negative‐charged patch (binds to synaptobrevin‐2), are highlighted by purple and blue shadows, respectively. Inset on the left shows the architecture of the intramolecule contacts of C_1_, C_2_B, and MUN domain. Inset on the right shows the interaction between the MUN‐D and synaptobrevin‐2 (Syb2) JLR.

The neuronal SNAREs syntaxin‐1, SNAP‐25, and synaptobrevin‐2 contain ~ 65‐residue sequences termed SNARE motifs that are able to form coiled coils [17, 19]. Syntaxin‐1 and synaptobrevin‐2 are anchored at the presynaptic membrane and synaptic vesicles, respectively, via the C‐terminal transmembrane region (TMR) which is connected to the SNARE motif by a short juxtamembrane linker region (JLR). SNAP‐25 lacks a TMR but is attached to the plasma membrane by palmitoyl chains bound to cysteine residues in an extended linker joining its two SNARE motifs. The neuronal SNAREs assemble into an intertwined and parallel four‐helical bundle called the SNARE complex via their SNARE motifs [17]. Crystal structure of the neuronal SNARE complex revealed that the core architecture contains 16 consecutive layers [20, 21], including layers −7 to −1 in the N‐terminal half, layers +1 to +8 in the C‐terminal half, and one central layer 0. These layers contain predominantly hydrophobic residues, except for the central layer 0, which comprises one arginine (R) and three glutamines (Q). The SNARE motifs are accordingly classified into Q_a_‐ (syntaxin‐1), Q_b_‐ (the first SNARE motif of SNAP‐25), Q_c_‐ (the second SNARE motif of SNAP‐25), and R‐SNARE (synaptobrevin‐2) [22, 23] (Fig. 1A). Phylogenetic and structural analysis of the SNARE motifs showed that the four SNARE subfamilies are diverged early during eukaryotic evolution and revealed a ‘Q_abc_R’ pattern for functional SNARE complexes e[23, 24, 25, 26, 27].

Unlike SNAP‐25 and synaptobrevin‐2, syntaxin‐1 possesses an N‐terminal regulatory sequence that is connected to the SNARE motif by a flexible linker region [28]; this regulatory sequence consists of an N‐terminal short stretch called the N‐peptide followed by an antiparallel three‐helix bundle called the H_abc_ domain. As with the SNARE motif, both the N‐peptide and the H_abc_ domain of syntaxin‐1 play indispensable roles in synaptic vesicle exocytosis [29, 30]. Although the N‐peptide and the H_abc_ domain are widely found in most of the Q_a_‐SNAREs [31, 32, 33, 34], their regulatory roles and mechanisms differ among different membrane trafficking systems [32, 35, 36, 37, 38].

## Neuronal SNARE complex assembly

**Fig. 2:**
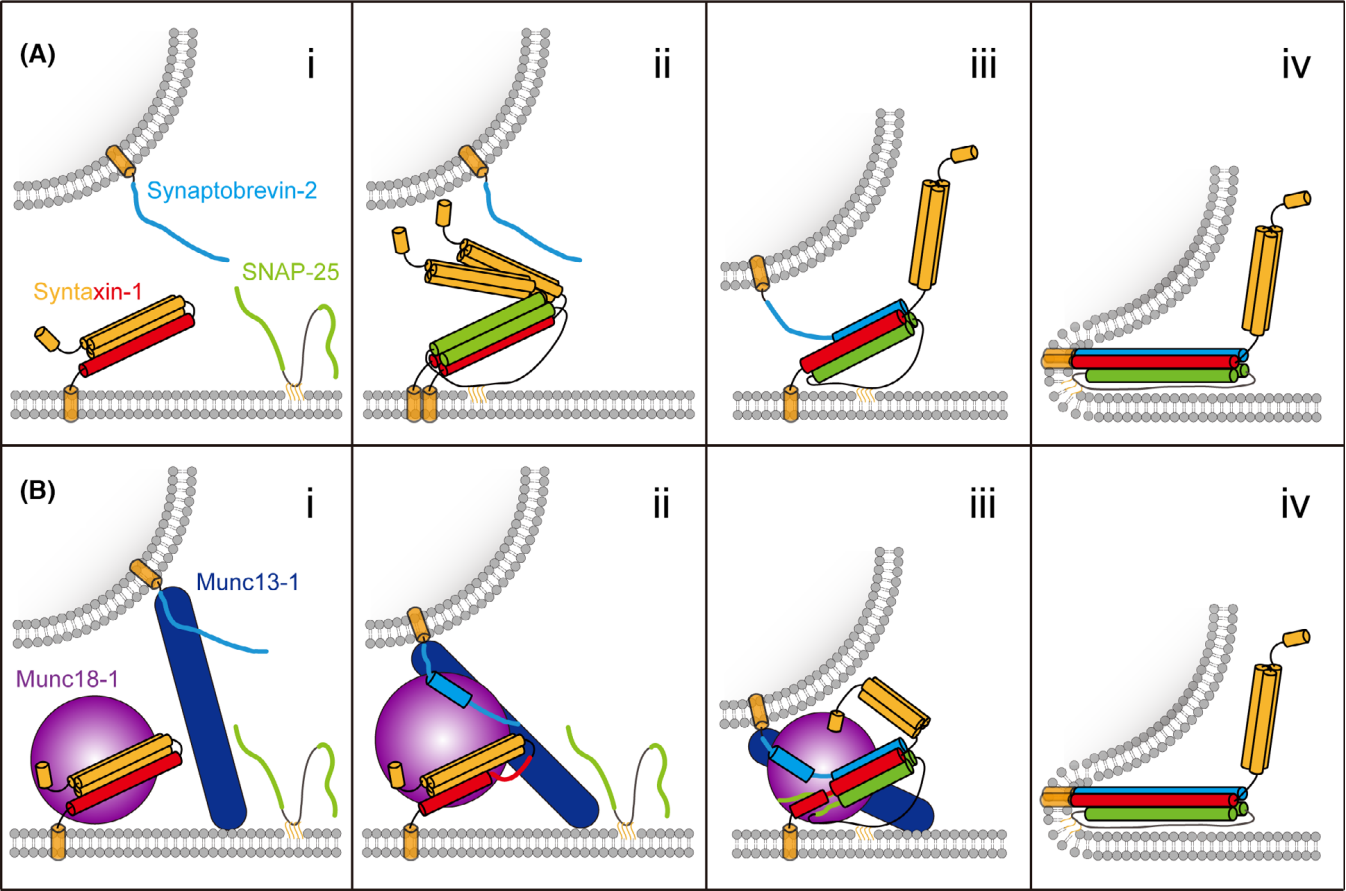
Models of SNARE‐mediated membrane fusion and synaptic exocytosis. (A) The zippering model with merely the three neuronal SNAREs. (i) At rest state, syntaxin‐1 adopts self‐inhibitory conformation; (ii) syntaxin‐1 fluctuates between closed and open conformations and is prone to form a 2 : 1 complex with SNAP‐25; (iii) synaptobrevin‐2 displaces one copy of syntaxin‐1 of the 2 : 1 complex; the N‐termini of the SNARE motifs nucleate together to promote complex assembly; (iv) zippering of the SNARE motifs transfers sufficient energy into the membrane thus catalyzing membrane fusion. (B) Munc18‐1 and Munc13‐1 synergistically organize neuronal SNARE complex assembly and synaptic exocytosis. (i) Munc18‐1 captures syntaxin‐1 into closed conformation; Munc13‐1 bridges the presynaptic membrane and synaptic vesicle to facilitate synaptic vesicle docking; (ii) Munc13‐1 interacts with Munc18‐1−syntaxin‐1 complex to induce conformational changes of the syntaxin‐1 linker region and Munc18‐1 domain 3a, leading to uncaging of the N‐terminus of the syntaxin‐1 SNARE motif and extension of Munc18‐1 domain 3a; in the meantime, Munc18‐1 interacts with the C‐terminal half of synaptobrevin‐2 with the assistance of the binding between Munc13‐1 and synaptobrevin‐2 JLR. This intermediate underlies a potential conformational state, namely the prefusion priming complex; (iii) the N‐termini of the SNARE motifs start to nucleate to produce a half‐zippered SNARE complex, which is organized by Munc18‐1 and Munc13‐1; (iv) full zippering of the SNARE motifs in response to calcium signal is accompanied by the interplay of complexins/synaptotagmin‐1 with the half‐zippered SNARE complex (not shown). The color schemes of the neuronal SNAREs are the same as in Fig. 1. Munc18‐1 and Munc13‐1 are colored in purple and navy blue, respectively.

The neuronal SNAREs undergo assembly–disassembly cycles to fulfill constant exocytosis of synaptic vesicles [14, 39, 40]. SNARE complex assembly is assumed to initiate with a contact of the N‐terminal ends of the SNARE motifs and proceeded with the association of the four SNARE motifs into the four‐helical bundle in a *trans*‐conformation that includes loose and tight intermediates underlying different priming states [41, 42, 43, 44, 45, 46] (Fig. 2A). The N‐ to C‐zippering of the *trans*‐SNARE complex can generate energy to overcome the repulsion of the opposite membranes thereby bringing the membranes into close proximity [47, 48, 49]. Subsequent assembly proceeding over the JLR and TMR of synaptobrevin‐2 and syntaxin‐1 is believed to transmit the energy into the membrane interface, leading to membrane fusion with the conversion of the SNARE complex from the *trans*‐ into a *cis*‐conformation in which the two TMRs are aligned in parallel in the plasma membrane [21, 50, 51, 52, 53, 54] (Fig. 2A). After membrane fusion, the *cis*‐SNARE complex is disassembled in an ATP‐driven manner by NSF and α‐SNAP [55, 56]. Once disassembled, free SNAREs are recycled for another round of synaptic vesicle fusion [57, 58, 59].

The SNARE zippering model embodies elegant simplicity and comprehensive unity suitable for most intracellular membrane trafficking systems. However, distinguished from most other membrane trafficking processes, synaptic vesicle exocytosis is extremely fast. Accompanied by large conformational change and high energy release [47, 48, 49], neuronal SNARE complex assembly go through multiple and complicated reactions [60], in which the detailed assembly kinetics and thermodynamics remain elusive. These, therefore, have raised many intriguing conundrums. First, more than 40 SNARE homologs have been identified in mammals, many of which are distributed in different cellular compartments and specific for different intracellular trafficking pathways [17, 27]. This raises an obvious question as to how the cognate SNAREs recognize each other to form a functional SNARE complex. Second, in the zippering model, syntaxin‐1 and SNAP‐25 are able to form heterodimeric complexes in the plasma membrane, which bind vesicle‐bound synaptobrevin‐2 to initiate *trans*‐SNARE complex formation (Fig. 2A). However, this assembly model faces several challenges: (a) syntaxin‐1 and SNAP‐25 are prone to form a 2:1 ‘dead‐end’ complex *in vitro*, in which a second copy of syntaxin‐1 occupies the position of synaptobrevin‐2 thus hindering the assembly kinetics of the SNARE complex [61, 62]; (b) isolated syntaxin‐1 prefers to assume a closed conformation in which the H_abc_ domain folds back to the SNARE motif to inhibit SNARE complex assembly [63]; and (b) *in vitro* lipid mixing driven by the neuronal SNAREs alone is strongly inhibited by NSF and α‐SNAP[64], owing to the disassociation of *trans*‐SNARE complexes and/or syntaxin‐1−SNAP‐25 heterodimeric complexes. These findings led to crucial questions about how nonproductive side reactions and kinetically trapped intermediates along the assembly pathway are prevented, and how the assembly is protected against disassembly factors. Third, the fusion kinetics obtained from *in vitro* reconstitution assay using the SNAREs alone is not comparable to that observed *in vivo*. In addition, previous studies reported that two complementary paired nucleic acid strands linked by the TMRs of syntaxin‐1 and synaptobrevin‐2 could mediate *in vitro* liposome fusion as well [65, 66]. Another study revealed that artificial coiled‐coil peptides linked by the JLRs and TMRs of the SNAREs could induce liposome fusion[67]. Therefore, it is apparent that liposome fusion catalyzed by the SNAREs alone could not factually reproduce membrane fusion *in vivo*.

The above considerations indicate a need for crucial factors to prime the SNAREs and organize SNARE complex assembly. A wealth of evidence has revealed that Munc18‐1 and Munc13s play a central function in synaptic vesicle priming via orchestrating SNARE complex assembly.

## Munc18‐1

Munc18‐1 is a member of the SM family proteins that have fundamental roles in most types of membrane trafficking processes from fungi to mammals [68, 69]. Loss of Munc18‐1 causes severe defects in neurotransmitter release of *Caenorhabditis elegans* motor neurons [70, 71, 72, 73] and of mice neocortex and neuromuscular synapse [74, 75, 76], revealing its critical role in synaptic vesicle exocytosis. Munc18‐1 knockout mice die immediately after birth. Analysis of the embryo brain lacking Munc18‐1 displayed massive neurodegeneration after assembly of the neuronal networks [74], indicating that Munc18‐1 is fundamental for the development of the nervous system. Munc18‐1 has been implicated to participate in synaptic vesicle docking, priming, and fusion. The diverse functions of Munc18‐1 depend, at least in part, on its capacity to bind the neuronal SNAREs with multiple conformations [13, 68, 69].

Crystal structures of Munc18‐1 bound to syntaxin‐1 revealed that Munc18‐1 forms an arch‐shaped architecture with three domains. Domain 3 is divided into two subdomains, that is, domain 3a and 3b. Domain 1 and domain 3a jointly create a central cavity to accommodate the H_abc_ domain and the SNARE motif of syntaxin‐1 in a closed conformation, which produces a high binding affinity between the two molecules (*K*_d_ ~ 1.4 nanomolar) [77, 78] (Fig. 1B). The closed conformation prevents syntaxin‐1 from being prematurely trapped by its cognate or noncognate Q_bc_‐ and R‐SNAREs during its translocation to the plasma membrane and renders vesicles suspended at the docking stage [76, 79]. Munc18‐1‐clamped syntaxin‐1 requires a transition from the closed to an open conformation to initiate SNARE complex assembly. This ‘closed‐to‐open’ transition appears to be a specialized feature of synaptic vesicle exocytosis, and the underlying mechanism will be discussed in more detail in the following sections.

The N‐peptide of syntaxin‐1 binds to domain 1 on the opposite side of the Munc18‐1 cavity (Fig. 1B), which is crucial for synaptic vesicle exocytosis [29, 30, 80]. This interaction not only assists Munc18‐1 to clamp the closed conformation of syntaxin‐1 [78] but also enhances binding of Munc18‐1 with the SNARE four‐helical bundle [81, 82, 83]. These data suggest that the N‐peptide binding is important for Munc18‐1 to assume diverse conformations available for syntaxin‐1 or the SNARE complex binding.

Moreover, domain 3a of Munc18‐1 can assume both ‘bent’ and ‘extended’ conformations. The bent conformation seen in Munc18‐1 bound to closed syntaxin‐1 is characterized as an inhibitory state of Munc18‐1, whereas the extended conformation represents an active state of Munc18‐1 which is accessible to synaptobrevin‐2 interaction [84, 85, 86, 87, 88, 89, 90] (Fig. 1B). This binding mode is conserved among various SM family proteins (e.g., Vps33 and Vps45) [36, 91]. Later studies showed that the majority of the SNARE binding layers of synaptobrevin‐2 (layer −4 to +6) were captured by the extended domain 3a of Munc18‐1, which is crucial to initiate SNARE complex assembly [92]. Furthermore, emerging evidence has indicated that Munc18‐1 is capable of binding to the SNARE four‐helical bundle. However, there is no consensus on the binding sites between Munc18‐1 and the SNARE four‐helical bundle, as evidence showed that the binding can be mediated by the cavity of Munc18‐1 [82, 84, 93, 94] or by domain 3a at the other side of the cavity [85, 87]. Despite lacking structural evidence, this binding mode is found in a variety of Munc18‐1 homologs and their cognate SNAREs [34, 95, 96, 97, 98], indicating a conserved function of Munc18‐1 in the final step of membrane fusion.

## Munc13‐1

As a member of the CATCHR family proteins, Munc13‐1 is a large multidomain protein highly enriched in the presynaptic active zones and conserved from invertebrates to mammals. Deletion of Munc13‐1 causes severe defects in neurotransmitter release, indicating its fundamental role in synaptic transmission [99, 100, 101, 102]. In addition, Munc13‐1 is important for dense‐core vesicle exocytosis in chromaffin cells, pancreatic beta cells, and neurons, demonstrating its essential function in the release of hormones and neuropeptides [103, 104, 105, 106]. Deletion of Munc13‐1 has no significant influence on the ultrastructure of synapse [101]. Loss of a mass of docked vesicles around the presynaptic active zones in Munc13‐1/2 knockout mice indicates that Munc13s are associated with the scaffold matrix formation of the active zones [107]. Similar phenotypes were observed in mice deficient in Rab3‐interacting molecules (RIMs) and RIM‐binding proteins (RIMBPs) [107, 108, 109, 110], and in Ca^2+^‐dependent activator protein for secretion (CAPS) [107] which belongs to the CATCHR family proteins as well [111].

The molecular architecture of Munc13‐1 contains multiple individual domains, including three C_2_ domains (C_2_A, C_2_B and C_2_C, respectively), a PKC‐like phorbol ester/diacylglycerol (DAG) binding C_1_ domain, a calmodulin‐binding motif (CaMb), and a central MUN domain (Fig. 1C). C_2_A resides in the N‐terminal part of Munc13‐1, which is a noncanonical C_2_ domain insensitive to Ca^2+^ [112]. C_2_A can form a homodimer and is able to bind the zinc‐finger (ZF) of RIMs to form a heterodimer [113, 114]. Munc13‐1, RIM, and Rab3 could form a ternary complex crucial for synaptic vesicle docking and priming [115]. Adjacent to C_1_, C_2_B binds Ca^2+^ and is competent for phosphatidylinositol (mainly phosphatidylinositol‐4,5‐diphosphate, PtdIns‐4,5‐P_2_) binding [116]. C_1_ and C_2_B serve as membrane anchors to ensure the binding between Munc13‐1 and the presynaptic membrane. Recent structural evidence and functional data have shown that C_1_ and C_2_B are tightly contacted and function in synaptic short‐term plasticity [117, 118, 119, 120]. CaMb is located between C_2_A and C_1_, which might modulate synaptic plasticity via calmodulin [121]. C_2_C is also Ca^2+^‐insensitive and found as a membrane‐binding module that preferentially interacts with synaptic vesicles. The function of C_2_C is indispensable, since deletion of C_2_C causes strong defects in synaptic vesicle exocytosis [122]. The MUN domain between C_2_B and C_2_C contains four subdomains (i.e., A, B, C, and D) that mainly consist of stacked α‐helices [123]. Sequence and structural analysis have indicated a remote but significant homology between the MUN domain and the subunits of various tethering complexes, such as the Exocyst complex, Dsl1 complex, COG complex, and GARP complex [123, 124, 125]. Hence, Munc13‐1 and the tethering factors may play a universal role in vesicle tethering and docking. Moreover, recent studies found that Munc13‐1 could form oligomers around the fusion pore *in vivo* and *in vitro* [126, 127, 128], indicating that Munc13‐1 serves as a scaffold and tethering factor in exocytosis.

Early studies demonstrated that the MUN domain could partially rescue neurotransmitter release in Munc13‐1 knockout mice, indicating that the MUN domain is the minimal module responsible for Munc13‐1 activity [129, 130]. The finding that syntaxin‐1 bearing the LE mutation that prefers an open conformation [63] can partially rescue release in *Caenorhabditis elegans unc13* nulls indicated that Munc13‐1 plays a role in opening syntaxin‐1 [131]. Moreover, the MUN domain has been found to mediate the transition from the closed Munc18‐1−syntaxin‐1 complex to the SNARE complex *in vitro* [132]. In addition, the crystal structure of a Munc13‐1 C_1_‐C_2_B‐MUN fragment indicated an intramolecular synergistic effect in which C_1_ and C_2_B may play roles in modulating the activity of the MUN domain [117]. Furthermore, a C_1_‐C_2_B‐MUN‐C_2_C fragment could bridge synaptic vesicles and the presynaptic membrane with at least two different orientations dependent on Ca^2+^ and presynaptic PtdIns‐4,5‐P_2_ and DAG levels, which is considered to be relevant to short‐term plasticity [117, 119, 120, 122].

In the past decade, important advances have been made in understanding the structural insights, intermolecular interactions, and functional mechanisms of Munc13‐1 and Munc18‐1 in synaptic vesicle exocytosis. In the next section, we will discuss the synergistic roles of Munc18‐1 and Munc13‐1 in organizing neuronal SNARE complex assembly.

## Organizing the SNAREs—synergistic roles of Munc18‐1 and Munc13‐1

Recent reconstitution experiments have illustrated a model in which Munc18‐1 and Munc13‐1 play vital functions in regulating SNARE complex assembly. This model suggests that the starting point of the assembly pathway is the closed Munc18‐1−syntaxin‐1 complex and its transition to the SNARE complex is highly regulated by Munc13‐1 (Fig. 2B) [64, 132]. It was previously observed that syntaxin‐1 together with SNAP‐25 is capable of aggregating in clusters with PtdIns‐4,5‐P_2_ in the presynaptic membrane under physiological condition [133, 134, 135]. The clusters are expected to involve a large number of syntaxin‐1−SNAP‐25 2:1 complexes unfavorable for synaptobrevin‐2 binding [61]. One of the exciting merits of this model is that the nonproductive intermediate—syntaxin‐1−SNAP‐25 2 : 1 complexes—could be prevented, as Munc18‐1 together with NSF and α‐SNAP effectively displaces SNAP‐25 from its complex with syntaxin‐1 in the membranes [64]. Actually, a 1 : 1 stoichiometry of the syntaxin‐1−SNAP‐25 complex is immensely beneficial for the efficient assembly of the SNARE complex. For instance, it was found that the ∆N‐SNARE complex (i.e., the 1 : 1 syntaxin‐1−SNAP‐25 complex bound to an N‐terminal truncated synaptobrevin‐2) facilitates synaptobrevin‐2 binding thus driving fast SNARE complex assembly and liposome fusion [44, 136].

Another virtue of this model is that Munc18‐1 and Munc13‐1 offer an effective protection mechanism for SNARE complex assembly. NSF and α‐SNAP could abolish fusion between syntaxin‐1−SNAP‐25 liposomes and synaptobrevin‐2 liposomes probably by destructing immature *trans*‐SNARE complexes and/or syntaxin‐1−SNAP‐25 complexes into individual SNAREs [64]. Intriguingly, fusion between Munc18‐1−syntaxin‐1 liposomes and synaptobrevin‐2 liposomes can robustly proceed in the presence of Munc13‐1, SNAP‐25, NSF, and α‐SNAP [64, 137, 138], suggesting that Munc18‐1 and Munc13‐1 protect SNARE complex assembly against disassembly by NSF and α‐SNAP. In addition, a syntaxin‐binding molecule tomosyn, which possesses an R‐SNARE‐like motif [139, 140, 141], was reported to arrest syntaxin‐1 and SNAP‐25 into a nonfusogenic product that precludes synaptobrevin‐2 entry [142, 143]. Interestingly, in the context of NSF and α‐SNAP, syntaxin‐1 is able to escape from tomosyn arrest and assemble into the Munc18‐1−syntaxin‐1 complex. Remarkably, Munc13‐1 can catalyze the transition from the Munc18‐1−syntaxin‐1 complex to the SNARE complex in a manner specific to synaptobrevin‐2 but resistant to tomosyn [144]. In this process, NSF and α‐SNAP assist Munc13‐1 to promote the conversion from tomosyn‐arrested syntaxin‐1−SNAP‐25 complex to the Munc18‐1−syntaxin‐1 complex, and finally to the functional SNARE complex. These findings illustrate a protection function of Munc18‐1 and Munc13‐1 in SNARE complex assembly and synaptic vesicle priming, consistent with the role of the HOPS tethering complex in protecting SNARE complex assembly and yeast vacuolar fusion against Sec18 and Sec17, the homologs of NSF and α‐SNAP in yeast [145].

As illustrated in the model, the transition from the Munc18‐1−syntaxin‐1 complex to the SNARE complex is catalyzed by Munc13‐1. Initiation of the transition requires activation of the closed Munc18‐1−syntaxin‐1 complex, which involves the opening of syntaxin‐1 and the extension of domain 3a of Munc18‐1. The catalytic site of Munc13‐1 responsible for opening of syntaxin‐1 positions at a hydrophobic pocket located in the middle portion of the Munc13‐1 MUN domain [123] (Fig. 1C). Disruption of the hydrophobic pocket (i.e., NFAA mutation) caused abrogation of MUN‐mediated transition from the closed Munc18‐1−syntaxin‐1 complex to the SNARE complex and led to strong defects in neurotransmitter release of *Caenorhabditis elegans* neuromuscular junction (NMJ) and mice cortical neurons, indicating that the NF pocket is central for Munc13‐1 catalytic activity [123, 146, 147]. The syntaxin‐1 linker region (bearing the LE sequence) between the H_abc_ domain and the SNARE motif was identified as the binding target for the Munc13‐1 NF pocket [146]. Biochemical and single‐molecule FRET data demonstrated that the Munc13‐1 NF pocket interacts with R151 and I155 in the linker region of syntaxin‐1, and this interaction specifically induces a conformational change of the syntaxin‐1 linker region [146, 148] (Fig. 2B). These data argue against the conventional assumption that syntaxin‐1 needs to be totally escaped from Munc18‐1 clamping to initiate SNARE complex assembly, suggesting that a local conformational change in the syntaxin‐1 linker region is sufficient to initiate SNARE complex assembly. On the other hand, domain 3a of Munc18‐1 is able to undergo ‘bent‐to‐extended’ conformational change during SNARE complex assembly [85, 86, 87, 88, 89]. Recent advances showed that binding of the Munc13‐1 MUN domain to domain 3a is required for MUN activity in opening of syntaxin‐1 [84] (Fig. 1B). Upon the opening of the syntaxin‐1 linker region by the MUN domain, domain 3a of Munc18‐1 is inclined to assume the extended conformation, which allows the N‐terminal end of syntaxin‐1 SNARE motif more accessible to nucleate with SNAP‐25 and synaptobrevin‐2 [84] (Fig. 2B). This cascaded reaction supports the N‐ to C‐zippering model of SNARE complex assembly [44, 45].

Efficient SNARE complex assembly requires N‐terminal nucleation of the SNARE motifs of SNAP‐25, synaptobrevin‐2 and syntaxin‐1 together. An attractive R‐SNARE−SM binding mode has been presented and attracted intense attention [91]. Based on this mode, later studies showed that the majority of the SNARE binding layers of synaptobrevin‐2 (layer −4 to +6) were captured by the extended domain 3a of Munc18‐1, with the N‐terminal layers of the SNARE motif being available for nucleation [92]. It is of note that the interaction between Munc18‐1 and synaptobrevin‐2 is quite weak, compared to their homologs Vps33 and Nyv1 in yeast, respectively [91]. A recent structural study reported a direct binding between the Munc13‐1 MUN domain and the synaptobrevin‐2 JMR [149] (Fig. 1C). This interaction enhances binding of synaptobrevin‐2 to the Munc18‐1−syntaxin‐1 complex [149]. Disruption of the interaction caused abrogation of MUN‐mediated transition from the closed Munc18‐1−syntaxin‐1 complex to the SNARE complex and led to strong defects in neurotransmitter release [149]. In addition, a more recent study identified an interaction between the bottom of domain 3a of Munc18‐1 and the SNARE motif of syntaxin‐1 [84]. It is likely that this binding enables Munc18‐1 to persistently associate with the SNARE motif of syntaxin‐1 during the transit of syntaxin‐1 from the Munc18‐1−syntaxin‐1 complex to the SNARE complex. Hence, Munc18‐1 and Munc13‐1 play crucial roles in SNARE assembly via priming syntaxin‐1 and synaptobrevin‐2.

The above interactions indicate the formation of a quaternary complex comprising Munc13‐1, Munc18‐1, syntaxin‐1, and synaptobrevin‐2 [147, 149] (Fig. 2B), which is available for efficient SNAP‐25 binding. SNAP‐25 is anchored on the presynaptic membrane via palmitoylation, which is naturally advantageous for SNARE nucleation. Notably, this proposed quaternary intermediate fully supports the half‐zippered mechanism of neuronal SNARE complex formation [45, 47], as truncation of the C‐half region of the second SNARE motif of SNAP‐25 (layer +1 to +8, residues 178−206) still supports Munc13‐1‐mediated transition from the closed Munc18‐1−syntaxin‐1 complex to the SNARE complex [149]. These data indicate that the C‐half region of the SNARE complex may be loosely packed along the assembly pathway guided by Munc18‐1 and Munc13‐1 (Fig. 2B), which is available for subsequent binding and regulation by complexins and synaptotagmins that are functionally related to Ca^2+^‐triggered fast fusion [150, 151, 152].

Moreover, Munc18‐1 and Munc13‐1 are implicated to protect SNARE complex assembly via preventing antiparallel binding of the SNAREs [153], and to promote artificial liposome fusion within 500 milliseconds under single‐vesicle level *in vitro* [154]. These data reinforce the physiological relevance of Munc18‐1 and Munc13‐1 in synaptic vesicle exocytosis. Altogether, the present data convey a fundamental model whereby Munc18‐1 and Munc13‐1 synergistically organize neuronal SNARE complex assembly and synaptic vesicle exocytosis.

## Homologs of Munc18‐1 and Munc13s in different membrane trafficking systems—universality and specificity

SNARE complex assembly is regulated by SM proteins and multisubunit tethering complexes (MTCs) to ensure cargo delivery to proper target organelles or the plasma membrane [124, 155, 156]. Despite being distributed in different subcellular locations across diverse eukaryotic cells, all known SNAREs have their cognate SM proteins (Table 1); and different MTCs also display functional cooperations with specific SM proteins and related SNAREs (Table 1).

**Table 1:** Summary of the interactions of MTCs, SNAREs, and related SM proteins.

MTCs^a^	SNARE interactions^b^	Related SM protein	SM‐related Q_a_‐SNARE	SM:Q_a_‐SNARE binding mode			
				N‐peptide	H_abc_ domain	SNARE motif	Four‐helical bundle
Dsl1 (3)	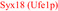 [157, 158 ] 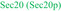 [157] 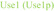 [157] 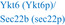 [159]	Sly1 (Sly1p)	Syx18 (Ufe1p)	•[33]	?	‐[33]	•[95]
TRAPPI (7)	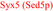 [160]	Sly1 (Sly1p)	Syx5 (Sed5p)	•[161]	?	‐[33]	•[34]
TRAPPII (10)	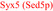 [160]	Sly1 (Sly1p)?	?				
TRAPPIII (8)	?	?	?				
COG (8)	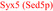 [162, 163, 164, 165] 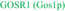 [166, 167, 168] 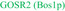 [163, 165] 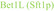 [169] 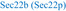 [170]	Sly1 (Sly1p)	Syx5 (Sed5p)	•[161]	?	‐[33]	•[34]
GARP (4)	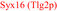 [171, 172, 173] 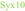 [174] 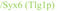 [175, 176] 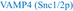 [171, 172]	Vps45 (Vps45p)	Syx16 (Tlg2p)	•[36]	•[36]	•[36]	•[96]
CORVET (6)	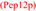 [177, 178] 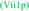 [179] 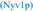 [91]	Vps33 (Vps33p)	? (Pep12p)				
HOPS (6)	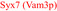 [37, 91, 180] 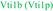 [179] 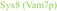 [180] 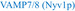 [91]	Vps33 (Vps33p)	Syx7 (Vam3p)	lacking[31]	•[181]	•[91]	•[97]
Exocyst (8)	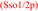 [182, 183] 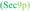 [184, 185] 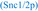 [183]	(Sec1p)	(Sso1/2p)	(lacking)[31]	(‐)[98]	(‐)[98]	(•) [98]
nCATCHR (2?)	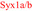 [132, 146, 186, 187, 188] 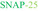 [189] 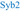 [149]	Munc18‐1	Syx1a/b	•[77]	•[77]	•[77]	•[94]

‘•’ Indicates direct binding between the motif/domain of Q_a_‐SNARE and SM protein; ‘‐’ indicates no binding between the motif/domain of Q_a_‐SNARE and SM protein. Syx, syntaxin; Syb, synaptobrevin.

^a^ Values in the parentheses indicate the subunits of MTCs in *Homo sapiens* and *Saccharomyces cerevisiae*.

^b^ Q_a_, Q_b_, Q_c_, and R‐SNAREs are indicated by red, green, light green, and blue, respectively. Values out of parentheses and in the parentheses indicate mammalian and *Saccharomyces cerevisiae* homologs, respectively.

In eukaryotic cells, seven MTCs have been identified, namely the Dsl1 complex [190], the TRAPP complex [191], the COG complex [192], the GARP complex [193], the CORVET complex [194], the HOPS complex [195], and the Exocyst complex [196], respectively, all of which participate in a variety of intracellular membrane trafficking pathways [156]. Despite differences in overall architecture and subunit composition, a subset of MTCs (the Dsl1, COG, GARP, and Exocyst complex) was characterized as the members of the CATCHR family proteins, in which the subunits of these complexes are composed of stacked long helical rods [124]. By contrast, the TRAPP, CORVET, and HOPS complex have different assembly and architecture [197, 198]. Different from other MTCs, the CORVET and HOPS complex involve the SM protein Vps33 as a subunit [194, 195]. The architecture of the Munc13‐1 MUN domain is similar to a variety of the CATCHR subunits such as Sec6 (Exocyst), Tip20 (Dsl1), COG5 (COG), and Vps54 (GARP) [124, 125]. Besides, CAPS contains a SNARE binding domain (DAMH), which is structurally similar to the CATCHR subunits and exhibits specific interaction with the Munc13‐1 MUN domain [186]. Therefore, it is expected that Munc13s and CAPSs might constitute a novel MTC—the neuronal CATCHR—to mediate synaptic vesicle exocytosis. It is believed that MTCs are involved in specific recognition of intracellular organelles and membranes by interacting with Rab GTPase [156]. Plenty of literature has reported that MTCs could interact with cognate SNAREs and SM proteins as well (summarized in Table 1). These interactions are crucial for recruiting the fusion components to the fusion sites [124, 156]. In addition, the HOPS complex was found to compete with the disassembly machinery (Sec17 and Sec18) and protect the prefusion SNARE complex from premature disassembly [145]; consistently, Munc13‐1 and Munc18‐1 were found to protect *trans*‐SNARE complex assembly in the context of Sec17−Sec18 homologs NSF−α‐SNAP [64, 137, 138]. Nevertheless, there still lack clear evidence whether other MTCs retain this function or not. Hence, the convergence of the function of MTCs is still limited among diverse membrane trafficking pathways.

Deletion of SM proteins invariably causes severe defects in membrane fusion (reviewed in [124, 155, 156]). As discussed above, a common characteristic of all SM proteins in membrane fusion is related to their function in templating SNARE complex assembly [34, 36, 84, 91, 92, 199, 200]. In spite of that, it is of note that there is substantial divergence in the binding between SM proteins and the SNAREs. SM proteins directly control the activity of Q_a_‐SNAREs with diverse binding modes (Table 1). For instance, Sly1p interacts with the N‐peptide to loosen the closed conformation of Sed5p and accelerate SNARE complex formation [201]; Vps45p rescues oligomeric Tlg2p into monomeric open conformation via interaction with the N‐peptide [36]; Vps33p interacts with both the H_abc_ domain and the SNARE motif of Vam3p to template SNARE complex formation [97, 181]; Sec1p binds to the assembled SNARE four‐helical bundle (Sso1p‐Sec9p‐Snc2p) with an unrevealed mechanism that likely involves opening of Sso1p [98]; Munc18‐1 captures syntaxin‐1 into the closed conformation and the opening of syntaxin‐1 requires catalysis by Munc13‐1 [132]. These diverse binding and activation modes indicate multiple mechanisms of SM proteins in regulating Q_a_‐SNAREs. Essentially, all SM proteins are implicated to interact with the assembled SNARE four‐helical bundle (summarized in Table 1), which not only facilitates SNARE complex assembly but potentially protects partially assembled SNARE complex from disassembly.

The neuronal MTC−SM shares a variety of common features with other ancient MTC−SMs, including the interplay between SM proteins and the N‐peptide of Q_a_‐SNAREs; the function to template and secure SNARE complex assembly via binding to Q_a_‐ and R‐SNARE simultaneously. Interestingly, the mode that Munc18‐1 traps syntaxin‐1 into the inactivated conformation has not been found in other membrane trafficking systems. It is conceivable that the neuronal system specifically evolves complementary mechanisms that involve the activation of the closed Munc18‐1−syntaxin‐1 complex by Munc13‐1. Altogether, it is likely that Munc13‐1 and Munc18‐1 inherit some fundamental roles of the ancient MTC−SMs and evolve specialized features that are highly adapted to synaptic vesicle exocytosis.

## Future perspective

In this review, we have summarized a variety of important advances toward understanding how Munc18‐1 and Munc13‐1 regulate SNARE complex assembly. However, fundamental questions still remain. First, there still lacks high‐resolution structural models of the whole release machinery that includes Munc18‐1 and Munc13‐1. This is a rather tough task since many interactions among these components are transient and weak, hindering the researchers from obtaining accurate structural models with atomic resolution. Single particle cryo‐electron microscopy (cryo‐EM) [202, 203] and *in‐situ* cryo‐electron tomography (cryo‐ET) [204] might be powerful tools to solve this issue. In addition, most present reconstitution studies still could not factually reproduce the fast kinetics of Ca^2+^‐triggered synaptic exocytosis. This may arise because additional components including many tethering and docking factors need to be included in these assays. Moreover, the presynaptic active zone is composed of a large number of macromolecules which create a crowded environment [4, 6]. Macromolecular crowding and liquid‐liquid phase separation (LLPS) might affect multiple reactions such as SNARE complex assembly and Ca^2+^‐triggered fusion [205, 206, 207, 208]. Hence, improvement of reconstitution methods that can reproduce the fusion events *in vitro* is necessary to better elucidate the molecular mechanism of synaptic vesicle fusion. Finally, there still exists unrevealed mechanisms of Munc13‐1 and Munc18‐1 in protecting *trans*‐SNARE complex assembly, in the terminal stage of membrane fusion, and in short‐/long‐term plasticity. Future studies remain challenging in fully resolving the fundamental molecular mechanism of synaptic vesicle exocytosis and the working principle of human brain.

## Conflict of interest

The authors declare no conflict of interest.

## Author contributions

SW and CM conceived and designed the project, and SW and CM wrote the paper.
